# Levofloxacin Cures Experimental Pneumonic Plague in African Green Monkeys

**DOI:** 10.1371/journal.pntd.0000959

**Published:** 2011-02-08

**Authors:** Robert Colby Layton, William Mega, Jacob D. McDonald, Trevor L. Brasel, Edward B. Barr, Andrew P. Gigliotti, Frederick Koster

**Affiliations:** Lovelace Respiratory Research Institute, Albuquerque, New Mexico, United States of America; Faculté de Médecine, Université de la Méditerranée, France

## Abstract

**Background:**

*Yersinia pestis*, the agent of plague, is considered a potential bioweapon due to rapid lethality when delivered as an aerosol. Levofloxacin was tested for primary pneumonic plague treatment in a nonhuman primate model mimicking human disease.

**Methods and Results:**

Twenty-four African Green monkeys (AGMs, *Chlorocebus aethiops*) were challenged via head-only aerosol inhalation with 3–145 (mean = 65) 50% lethal (LD_50_) doses of *Y. pestis* strain CO92. Telemetered body temperature >39°C initiated intravenous infusions to seven 5% dextrose controls or 17 levofloxacin treated animals. Levofloxacin was administered as a “humanized” dose regimen of alternating 8 mg/kg and 2 mg/kg 30-min infusions every 24-h, continuing until animal death or 20 total infusions, followed by 14 days of observation. Fever appeared at 53–165 h and radiographs found multilobar pneumonia in all exposed animals. All control animals died of severe pneumonic plague within five days of aerosol exposure. All 16 animals infused with levofloxacin for 10 days survived. Levofloxacin treatment abolished bacteremia within 24 h in animals with confirmed pre-infusion bacteremia, and reduced tachypnea and leukocytosis but not fever during the first 2 days of infusions.

**Conclusion:**

Levofloxacin cures established pneumonic plague when treatment is initiated after the onset of fever in the lethal aerosol-challenged AGM nonhuman primate model, and can be considered for treatment of other forms of plague. Levofloxacin may also be considered for primary presumptive-use, multi-agent antibiotic in bioterrorism events prior to identification of the pathogen.

## Author Summary

*Yersinia pestis* is the causative agent of bubonic plague as well as a rare severe form known as primary pneumonic plague resulting from the inhalation of contaminated aerosols. The relative ease of aerosol preparation and high virulence makes *Y. pestis* a dangerous bioweapon. The current study describes the treatment of established pneumonic plague with the widely available, broad-spectrum fluoroquinolone antibiotic levofloxacin in a nonhuman primate model. African green monkeys inhaled a target dose of 100 lethal doses for 50% of animals (LD_50_) and were monitored for fever and vital signs by telemetry. Fever was the first sign of illness, correlating with bacteremia but preceding radiographic pneumonia, and initiated intravenous levofloxacin treatment in doses designed to mimic antibiotic levels achieved in humans. All animals treated with saline died and all animals completing 10 days of treatment survived, with resolution of high fever within 24–48 hours. We conclude that levofloxacin may be an appropriate broad-spectrum antibiotic for presumptive therapy in an aerosolized bioweapons attack and should be studied for treatment of bubonic plague.

## Introduction

*Yersinia pestis* is the causative agent of bubonic plague, initiated by the bite of an infected flea, and primary pneumonic plague (PPP, often called inhalational plague) resulting from the inhalation of aerosolized contaminated environmental dusts [1], [2]. *Y. pestis* is also one of the most dangerous bioweapons due to the relative ease of lethal aerosol preparation, high virulence, the rapidity of onset of symptoms and death by PPP, and its history of use as a bioweapon [3]. Naturally acquired PPP is relatively rare with few outbreaks in the developing world [4], [5], the risk of person-to-person dissemination is low [6], and few detailed reports describe the evolution of the human disease [7], [8], [9].

Development of vaccines and therapeutics for plague can not utilize human trials either in natural or intentional infections, and must rely on animal models. The U.S. Food and Drug Administration (FDA) “animal rule” (21 CFR Part 314) permits approval of therapeutics and vaccines based on testing in appropriate animal models. The mouse model [10],[11],[12] and rat model [13] of pneumonic plague bears multiple similarities to the human disease, including a brief anti-inflammatory incubation period followed by the rapid evolution of the pro-inflammatory fulminant disseminated disease. The molecular arsenal secreted by *Y. pestis* is well characterized [14] and appears to mediate the anti-inflammatory phase in the lung [15], [16]. Potential antibiotic therapies have been screened in the mouse model [17], but valid extrapolation of efficacy from mice to humans is not yet established.

Nonhuman primates have been known for decades to be highly susceptible to the pneumonic form of plague [18], [19], [20] and have been considered extensively for their value as animal models of pneumonic plague [21]. The African Green monkey (AGM) is also highly susceptible to *Y. pestis* infection by the aerosol route [20]. The AGM may have several advantages over the cynomolgus macaques in that telemetered fever above 39°C is the first and uniform sign of systemic disease following aerosolized *Y. pestis* challenge, and there appears to be less individual variation in innate resistance among the AGMs [22].

Identifying the optimal antibiotic for treatment of pneumonic plague faces several challenges. First the disease presents with non-specific symptoms of fever and pneumonia until late stages when hemoptysis suggests a diagnosis other than community-acquired pneumonia (CAP). Syndromic surveillance may not detect an outbreak within a few days of a bioterrorism event, and laboratory diagnosis may be delayed [23], [24], [25]. Second, the antibiotic must be widely available in all hospitals and established as a drug-of-choice not only for bacterial pneumonia but also for multiple biothreat agents including anthrax [26], [27]. Third, the antibiotic should ideally have excellent oral bioavailability, so that use of oral antibiotic could be used if a massive bioterrorism event taxed hospital facilities. The currently recommended antibiotic to treat plague, gentamicin, is not widely used in CAP unless *Pseudomonas* species are suspected. However, levofloxacin satisfies the second and third criteria since it is efficacious and widely used for CAP [28], [29].

The objective of this study was to evaluate the efficacy of intravenous treatment with levofloxacin following a lethal aerosol challenge to *Y. pestis* CO92 in the AGM model. Treatment was initiated after the onset of fever in order to use a readily available clinical marker of established disease rather than rely on a marker for bacteremia or other indicator of disseminated disease. Even though targeted levels of plasma antibiotic concentrations were not uniformly met, all treated AGMs survived established pneumonic plague, and resolution of systemic signs was apparent after only 2–3 days of treatment.

## Materials and Methods

### Nonhuman primates

Wild-caught African Green monkeys (*Chlorocebus aethiops*) (Alpha-Genesis, Inc.) weighed 3–8 kg and were at least 2 yrs old. All procedures were conducted under protocols approved by the Institutional Animal Care and Use Committee in Lovelace Respiratory Research Institute facilities accredited by the Association for Assessment and Accreditation of Laboratory Animal Care International (AAALAC). Animals were individually housed in stainless steel cages with wire mesh bottoms, in rooms on a 15/9-h light/dark cycle at temperature between 20–25°C and relative humidity between 11–73%. Diet was Harlan Teklad Certified 20% Monkey Diet 2050C (Harlan-Teklad) twice daily, supplemented with treats, with *ad libitum* municipal tap water.

Animals were conditioned to a restraint collar, poles, restraint chairs, and limb restraints. Radiotelemeters (Model T30F, Konigsberg, Inc.) were implanted subcutaneously in the left abdominal wall for continuous monitoring of body temperature, intrathoracic pressure, respiratory rate, heart rate, and electrocardiographic traces. Venous access catheters (Broviac, Cohorts 1 and 2; or Hickman dual-port, Cohort 3) were inserted in the right femoral vein, tunneled through the right flank and back, emerging through the skin of the upper mid-back and protected by a jacket [30]. No study animals had received systemic antibiotics within 28 days prior to aerosol exposure with *Y. pestis* strain CO92 nor topical mupirocin ointment within 14 days of aerosol exposure. Animals were moved into the Animal Biosafety Laboratory-3 at least 1 week prior to aerosol challenge with *Y. pestis* to permit acclimatization and obtain baseline values for telemetry measurements.

Twenty-six AGMs in three cohorts were randomized into treatment groups. Two animals were removed from Cohort 2, one prior to infectious challenge due to health reasons and one after challenge due to initiation of treatment prior to becoming febrile. The subsequent analysis included the remaining 24 animals. Animals were randomized into test groups using a computerized data acquisition system (Path-Tox 4.2.2; Xybion) based on body weights and randomized into exposure order using Microsoft Excel's random number generator.

### Challenge pathogen

*Y. pestis* strain CO92 was originally isolated in 1992 from a person with a fatal case of pneumonic plague [8] and was supplied by C.R. Lyons at the University of New Mexico. All work done was performed under Biosafety Laboratory-3 conditions. For each cohort exposure, one working stock cryovial of *Y. pestis* was removed from frozen storage, thawed, and used to inoculate five tryptose blood agar base (TBAB)+yeast extract slants. After incubation at 28±2°C for 72±8 h the slants were washed with 1% peptone, combined and centrifuged at 4100 rpm at 5±3°C for 25±5 min. The cell pellet was suspended in 1% peptone and the optical density at 600 nm (OD_600_) was determined. The bioaerosol sprays were prepared in brain heart infusion broth (BHIB) from the suspended centrifuged culture based on the OD_600_ and a previously prepared concentration/OD curve. The suspension was adjusted to achieve the target aerosol exposure dose of 100±50 LD_50_ doses or approximately 35,000 cfu of *Y. pestis* [Pitt MLM, DN Dyer, EK Leffel, et al. Ciprofloxacin treatment for established pneumonic plague in the African Green Monkey. Abstract B-576, 46^th^ICAAC meeting, San Francisco, CA, September 28, 2006].

### Inhalation challenge

After fasting overnight the animals were anesthetized with 2–6 mg/kg Telazol 15 min prior to aerosol exposure and baseline radiographs (Study Day 0). The exposure system consisted of a head-only exposure unit contained in a Class 3 biosafety glovebox [31] and previously described in our laboratories [32], [33]. Real-time plethysmography (Buxco) measuring respiratory frequency, tidal volume, and minute volume targeted an inhaled volume of 5 L, with actual exposure times ranging from 10–15 min. Suspensions of *Y. pestis* strain CO92 were nebulized in a Collison nebulizer (MRE-3 jet, BGI, Inc.), and delivered to the freely breathing anesthetized AGMs. The bacteria-containing aerosol was sampled directly into an all glass impinger (AGI; Ace Glass, Inc.) drawn from the head-only exposure apparatus downstream from the primate's nares and bacteria concentrations were confirmed by quantitative bacterial culture and purity was assessed by colony morphology. The target particle size was 1–3 µm, determined using an Aerodynamic Particle Sizer Spectrometer (Model 3321, TSI, Inc.; Cohorts 1 and 2) or a GRIMM Portable Aerosol Spectrometer Model 1.109 (Cohort 3) for 0.5–20-µm particles. The mass mean diameter of the aerosols was determined to be 1.9–2.4 µm (1.32–2.80 geometric standard deviation). Aerosolized pathogen dose was calculated using the following formula: Dose = (C×V), where C is the concentration of viable pathogen in the exposure atmosphere, and V is the volume inhaled.

### Post-challenge antibiotic administration

Levofloxacin (Levaquin Injection Premix in Single-Use Flexible Containers as 5 mg levofloxacin/mL 5% dextrose; Ortho-McNeil, Inc.) or the control solution (5% dextrose in water, D_5_W) was infused into the femoral vein catheter by syringe pump over 30±5 min. Infusions were initiated within 6 h of the appearance of telemetered fever defined as a mean temperature ≥39°C for more than 1 h. Because previous studies demonstrated a clearance of levofloxacin approximately three times more rapid in rhesus macaques than in humans [10], daily levofloxacin infusions were dosed at 8 mg/kg body weight, followed by 2 mg/kg administered 12.0±0.5 h later. Infusions of levofloxacin or D_5_W were continued until death, moribund euthanasia, or 20 infusions had been completed.

### Monitoring procedures

Clinical observations were made twice daily cage-side noting activity, posture, nasal discharge, sneezing, coughing, respiratory characteristics, ocular discharge, inappetance/anorexia, stool characteristics, seizures, neurologic signs, or other abnormalities. Body weights were measured within 1 week prior to aerosol exposure, on the day of aerosol challenge, and at necropsy.

Implanted T30F telemetry devices continuously monitored body temperature, respiratory rate, heart rate, and electrocardiogram. Temperature, respiration signal, and ECG was recorded by CA Recorder software (D.I.S.S., LLC) every 5 min and averaged for hourly values by VR^2^ software (D.I.S.S., LLC). Respiratory rate was recorded by intrapleural pressure changes. Heart rate was recorded by the software counting R waves per minute.

The decision for euthanasia was based on the development of at least two moribund criteria: >60 respirations/min or deep labored breathing; abnormal repolarization signals (persistently inverted T waves or depressed ST segment); seizures; falling off perch; unresponsive to stimulation; refusal to eat offered food. The Principal Investigator or staff veterinarian making decisions regarding euthanasia was blinded to the animal's treatment group.

A digital chest X-ray was taken at the time of anesthesia prior to aerosol exposure on Day 0, on Day 5 for animals necropsied for moribund disease or surviving to that point, and on Day 28 for animals surviving in the first two cohorts. Radiographs were qualitatively reviewed by a veterinary radiologist (Veterinary Imaging Center of South Texas) who was blinded to treatment group and stage of disease.

For levofloxacin plasma concentrations a sample of venous blood was drawn 10–30 min before the onset of infusions number 3, 6, and 19 (trough level) and 5–15 min (peak level) after the termination of infusions number 1, 3, 6, and 19. The plasma was centrifuge-filtered through a 0.2-µm Nanosep MF centrifugal filter (Pall Corp.) at 13,000×g for 40 min and extracted in 4 ml dichloromethane containing 250 µL KH_2_PO_4_ 70 mM∶NaHPO_4_7H_2_O 80mM, 2∶3 v∶v. After phase separation the dichloromethane was evaporated, and the residue was reconstituted with 100 µL acetonitrile∶pure water, 1∶1 v∶v with 0.1% formic acid. Chromatographic separation was conducted with an Agilent 1100 HPLC with a Discovery HS F5 (Supelco #56700-U) column. The fluorescence detector was set at excitation wavelength of 296 nm and emission wavelength of 504 nm. Data was processed using Varian Galaxie Chromatography Data System software version 1.8.505.5. The lower limit of quantification was established at 30 ng/ml and the method met pre-determined performance criteria for selectivity, accuracy, precision, recovery, calibration curve, and dilutional linearity.

For quantitative bacteriology daily on Days 2–6, and on Days 14 and 28, venous blood (target volume of 0.5–1.5 mL) was collected percutaneously from the femoral vein through a site washed three times with povidone iodine, transferred to an EDTA tube, and three log_10_ dilutions plated. To increase sensitivity, an undiluted 1-mL aliquot was inoculated into heart infusion broth w/ 1% xylose, incubated for up to 72 h and if growth noted, plated for confirmation of *Y. pestis*.

Serum for clinical chemistry was collected before exposure and on Days 2, 6, and 28 post challenge and analyzed using a Hitachi 911 Clinical Chemistry Analyzer (Roche Diagnostics) or a PMod Clinical Chemistry Analyzer. Whole blood for hematology was collected percutaneously from the femoral vein and transferred to a tube containing EDTA. One drop was smeared onto a glass slide for manual differential count and analyses were made using an Advia 120 (Bayer Corporation, Diagnostic Division).

### Pathology

Moribund or end-of-study animals were anesthetized with intramuscular 10 mg ketamine/kg body weight and euthanized by intravenous Euthasol. Tissues, including lung, tracheobronchial lymph nodes, liver, spleen, and brain, were collected for quantitative bacteriology and histopathology. Lung lobes were gently inflated with 10% neutral-buffered formalin (NBF) to approximate normal volume prior to immersion fixation. Tissues sections were fixed in NBF, cut 4–6 µm thick, mounted on slides, and stained with hematoxylin and eosin.

### Statistics

Summary statistics (e.g., means, standard deviation, charts, graphs, etc.) were calculated for quantitative parameters (BioSTAT Consultants, Portage, MI). Survival was the primary endpoint and was examined by Fisher's exact test. Analyses of secondary endpoints were performed as repeated measures ANOVA (SAS). For all analyses, a P value of ≤0.05 was considered to be a significant difference.

## Results

### Inhalation exposure and treatment effect

**Figure 1:**
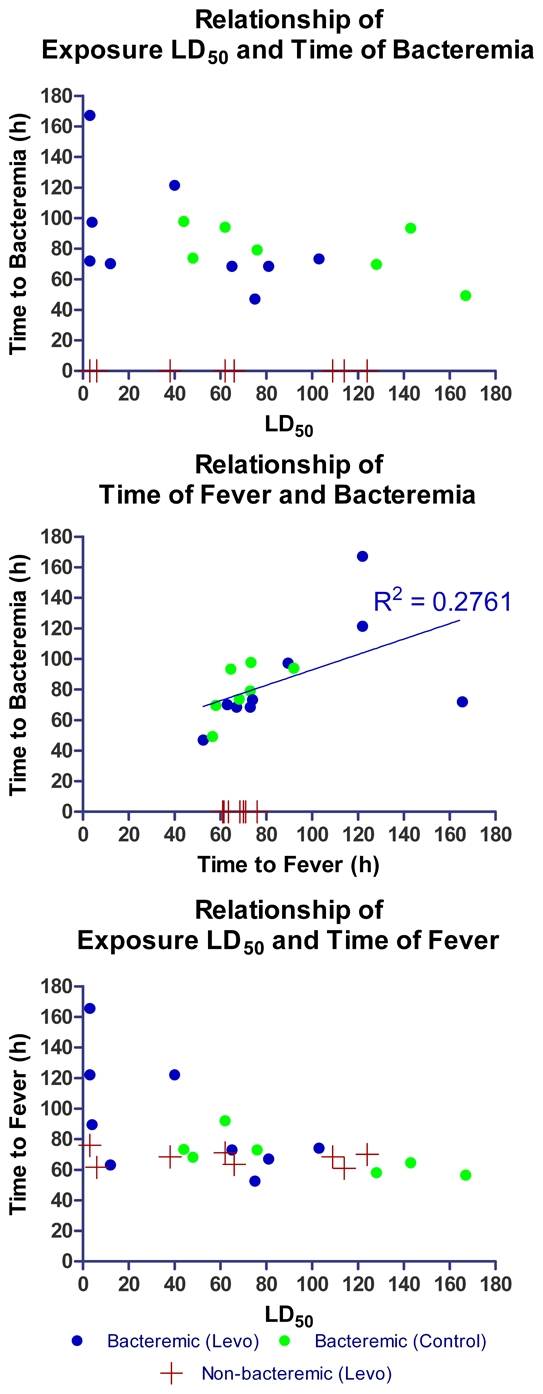
Comparison of time to bacteremia, LD_50_, and time to fever. Relationships between exposure dose in multiples of LD_50_ inhaled *Y. pestis* and onset of fever detected by telemetry and onset of bacteremia detected by culture of once-daily femoral artery blood collections. Time intervals of animals with negative blood cultures are indicated by red crosses. No statistically significant relationships are found between exposure dose, bacteremia and fever onset.

AGM were exposed to aerosolized *Y. pestis* CO92 in three separate cohorts. The estimated group mean (±SD) -delivered LD_50_ doses were 74 (31.0) in the first cohort, 124 (10.5) in the second cohort, and 22 (23.1) in the third cohort, with a range over all animals from 3–145 LD_50_ doses (Figure 1). All seven control animals succumbed to the challenge with *Y. pestis* CO92 and were moribund euthanized or died before euthanasia could be performed on Days 4 or 5 following challenge. All 16 AGMs treated with levofloxacin for 10 days survived until planned euthanasia on Day 28. One animal (Y160) began treatment on day 3 pe but was euthanized on Day 9 was after one day of vomiting and inability to retain food. Blood culture was positive for *Y. pestis* at initiation of treatment on Day 3 but subsequent blood cultures on Days 4–7 and tissues collected at necropsy were negative. Histopathology of the stomach revealed necrosis of the gastric epithelium and no evidence of active *Y. pestis* infection in other organs. While there was no evidence for treatment failure in this animal, in an intention-to-treat analysis (including Y160) the difference in survival was statistically significant at p<0.001, (Wilcoxon).

In cohort 3 seven of the eight treated animals were delivered doses of aerosolized *Y. pestis* less than that received by the two controls. If the animals in Cohort 3 are removed from analysis, the difference in survival between treatment and control remains significant (p<0.001).

### Bacteremia

**Figure 2:**
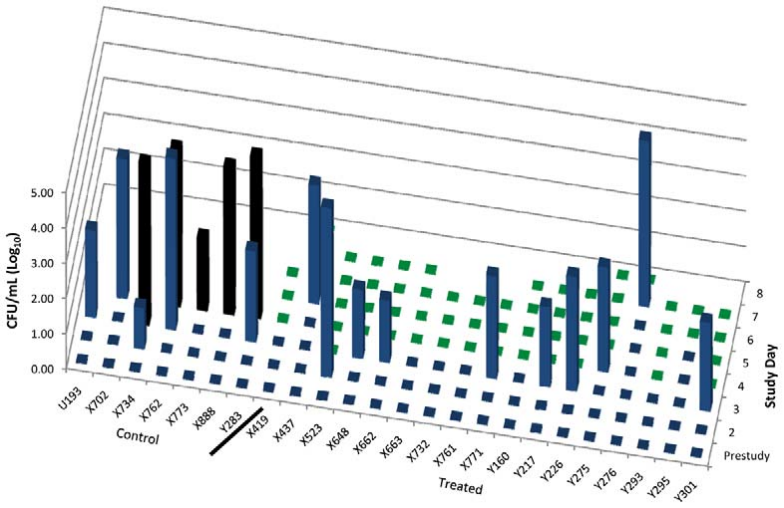
Pre-infusion and post-infusion bacteremia in controls and treated AGM. Quantitative bacteremia prior to and after initiation of levofloxacin (treated, N = 16) or D_5_W (control, N = 7) infusions. Blue bars indicate level of bacteremia prior to infusion. Black bars indicate level of bacteremia post-infusion for control animals and green bars for treated AGM. Nine AGM did not have bacteremia detected prior to infusion with D_5_W (N = 2) or levofloxacin (N = 7).

Among the 7 controls only 5 were bacteremic prior to onset of fever and infusions of D_5_W with bacterial loads ranging from 1.2–4.9 log10 CFU/mL (**Figure 2**). All 5 controls tested more than 12 h after onset of infusons were bacteremic with bacterial loads of 2.5–5.5 log10 CFU/mL. Among the levofloxacin-treated animals, 13 of 17 (76%) had *Y. pestis* bacteremia detected prior to or at the onset of fever and infusions with levels ranging from 1.8 to 4.9 log10 CFU/mL. No bacteremia was detected after onset of levofloxacin infusions up through day 7 post-exposure, or a total of 60 post-treatment samples.

Lung tissues from levofloxacin-treated animals lacked detectable *Y. pestis* at necropsy 11–17 days after termination of antibiotic infusions. Tissue pathogen loads in untreated animals ranged from 1.5×10^4^ to ≥3.0×10^5^ cfu/mL and tissue load was highest in the lungs and tracheobronchial lymph nodes up to 1.5×10^10^ cfu/g. There was no significant relationship between onset of fever and detection of bacteremia by once-daily sampling of whole blood, nor were there significant relationships between inhalation challenge dose and onset of fever or onset of detected bacteremia (**Figure 1**).

### Vital sign telemetry

**Figure 3:**
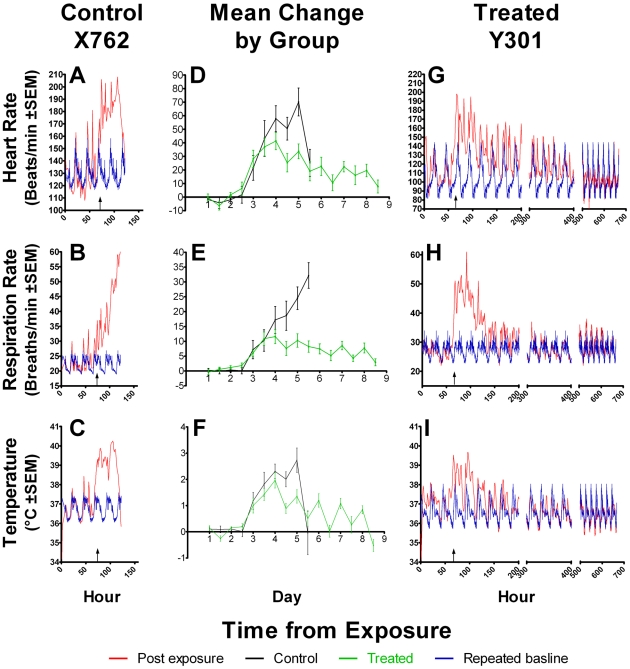
Heart rate, respiration rate and temperature for a control, group means, and a treated AGM. Telemetry data is expressed as mean values for each hour post-exposure for one control untreated animal X762 (A:heart rate, B:respiratory rate, C:body temperature) and for one treated animal Y301 (G: heart rate, H: respiratory rate, I: body temperature). Post-exposure data are in red, repeated baseline averages and SEM are shown in blue. Repeated baseline averages are shown to represent the animal's physiological measurement would be without disease at that time post exposure. Group means and standard deviation (error bars) were calculated as delta values (difference between baseline value and pe value at 00:00 and 12:00 of each day) for treated or control animals (D: heart rate, E: respiratory rate, F: temperature). Group means are pooled from all three cohorts.

The raw data were calculated as hour averages for temperature, heart rate and respiratory rate as illustrated in **Figure 3**. For statistical comparisons the data for each animal was calculated as change from the baseline (delta value) for that animal at that time of day. The means (standard deviations) of each treatment group for each vital sign recorded is illustrated in **Figure 3** **D, E and F**. The delta values at 12:00 and 00:00 (midnight) for each day are displayed to account for diurnal variation. Increases above baseline values for each animal in body temperature, heart rate, and respiratory rate were apparent during the Day 2 to Day 3 interval but for both the control and treated groups of animals significant increases (delta values) were not seen until Day 3 (approximately 72 h pe). By Day 4.5 the group mean increase in all three vital signs of the control group was greater than the group mean increases over baseline in the treated group. The apparent return to baseline body temperature in the control group at day 5.5 pe was due to moribund hypothermic animals at this time. The resolution of fever, tachycardia and tachypnea in the treated group was gradual over the next 4 days until day 9 when vital signs had returned to baseline (**Figure 3 G, H, I**). After the end of the 10-day treatment, the African Green monkeys were observed for an additional 11 to 16 days and telemetered temperature, respiratory rate, and heart rate revealed no evidence of clinical relapse.

### Radiology

**Figure 4:**
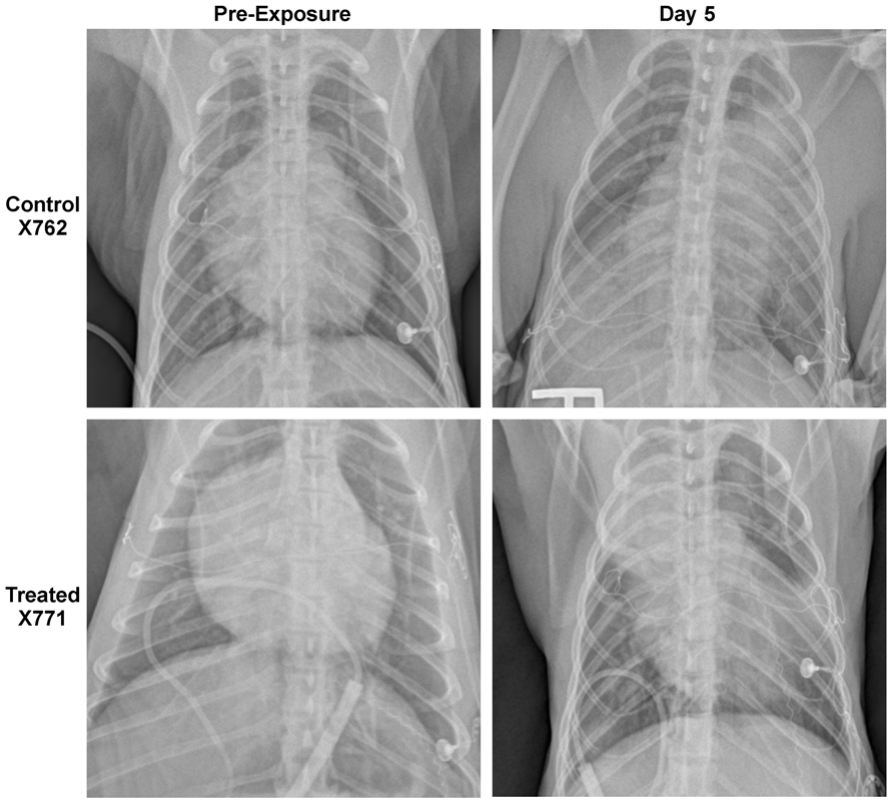
Chest radiographs of a control and treated animal prior to and 5 days after challenge. Chest radiographs obtained prior to challenge (left) and 5 days post exposure (right) for two macaques typical of findings in other animals, The untreated control animal (X762) displayed large infiltrates in the right caudal, left middle and left cranial lobes immediately prior to euthanasia for respiratory distress. The treated animal (X771) had initiated levofloxacin therapy 20 hours prior to pulmonary imaging on day 5 pe and displayed large infiltrates in the right cranial lobe and left caudal lobe, superior segment, with minor infiltrates in the left cranial lobe and right caudal lobe.

All pre-challenge radiographs showed no underlying disease. In all three untreated animals studied, large multilobar infiltrates correlated in location with palpable consolidation noted during necropsy. In general, however, the radiographs underestimated the extent of pneumonia found by gross pathology. In all nine animals treated with levofloxacin in Cohorts 1 and 2, chest radiographs on Day 5 revealed pulmonary infiltrates in one to four lobes. These radiographs were obtained approximately 120 h after aerosol challenge and up to 67 h after fever onset and antibiotic initiation, indicating that pneumonia was established in all treated animals (**Figure 4**). Pulmonary radiographs 28 days after challenge (15–17 days after termination of therapy) in five treated animals in Cohort 1 were normal without any apparent residual of previous pulmonary infection.

### Levofloxacin plasma concentrations

The levofloxacin levels measured in the first four samples taken in eight treated animals demonstrate intragroup consistency (**Table 1**). The peak (maximum concentration, C_max_) level after the first and second doses of 8 mg/kg body weight ranged from 2.4–4.6 µg/mL. Only 3 of the 16 infusions attained a level within two standard deviations of the target concentration of 6.2±1.0 µg/mL, yet all animals treated for 10 days survived the lethal aerosol challenge. All trough levels exceeded the MIC of the *Y. pestis* CO92 strain (0.03 µg/mL) [34].

**Table 1:** Plasma levofloxacin levels in cohort 3.

Infusion No.:	Peak after 1^st^ high dose		Trough before 2^nd^ high dose after 1^st^ low dose		Peak after 2^nd^ high dose		Peak after 2^nd^ low dose	
Dosage^a^:	8 mg/kg		2 mg/kg		8 mg/kg		2 mg/kg	
ID	Levo b	ΔT c	Levo	ΔT	Levo	ΔT	Levo	ΔT
Y160	2893.91	0:05	52.85	10:45	3016.46	0:08	1015.10	0:05
Y217	4514.94	0:06	60.50	10:50	3841.48	0:05	1304.52	0:05
Y226	3227.63	0:05	59.51	10:47	2475.65	0:09	1009.28	0:06
Y295	2428.36	0:10	35.53	11:16	3426.33	0:08	724.13	0:05
Y275	2582.17	0:05	117.33	10:23	3320.44	0:05	1445.88	0:05
Y276	4260.60	0:06	70.02	11:08	2909.78	0:05	1102.96	0:05
Y293	2688.83	0:05	95.97	10:49	2853.31	0:07	1084.79	0:07
Y301	4011.95	0:06	69.84	10:38	4552.10	0:05	945.10	0:08

a Dosage level of previous infusion prior to blood collection.

b Plasma levofloxacin concentration (ng/mL).

c Change in time (h∶m) from end of previous infusion.

### Clinical chemistry

Serum alanine aminotransferase (ALT), aspartate aminotransferase (AST), lactate dehydrogenase (LDH), and total bilirubin increased in control animals at euthanasia and treated animals on day 6, compared to pre-study values (data not shown, p<0.01) but there was no statistical difference in these values between the control and treated groups. Creatinine was elevated only in the control group. Treated animal values returned to pre-study values by the end of the study.

### Hematology

Total white blood cell count increased in the control group from 11.2±2.5×10^3^/dL(mean ± SD) to 41.0±32.9×10^3^/dL at euthanasia on day 5, compared to no increase in the treated group (baseline 9.5±2.4 compared to 12.0±4.4 at Day 6). Differences in neutrophil and monocyte countsbetween the two groups are consistent with a decrease in inflammation after approximately two days of antibiotic administration. The hematocrit increased in the control group (Day 5, 55±6.1) but not in the treated group (Day 6, 39.7±5.2) compared to baseline values for both groups (47.5±5.5).

### Histopathology

In the moribund control animals multiple lobes contained extensive parenchymal hemorrhage and marked fibrinosuppurative pneumonia. Findings included enlarged tracheobronchial lymph nodes, discolored liver and spleen without enlargement, an enlarged heart in two animals, and fluid on the brain of one animal. Septal histiolymphocytic infiltrates within the pulmonary parenchyma seen in over half of the levofloxacin-treated animals 28 days after challenge are consistent with resolution of pneumonia. No evidence of abscesses or neutrophilic alveolitis was found in any levofloxacin-treated animal 28 days after challenge.

## Discussion

This study demonstrated the efficacy of intravenous levofloxacin treatment to prevent death from lethal pneumonic plague in AGMs. All seven animals receiving intravenous D_5_W died while all 16 animals completing the 20-infusion course of levofloxacin survived until Day 28 post-exposure. Demonstration of efficacy depended on three features of the study design: selection of the AGM model, initiation of treatment for severe disease but prior to irreversible disease, and selection of the appropriate antibiotic and dosing schedule.

### Model selection

The AGM model mimics human disease in most respects including precipitous course of disease following a brief asymptomatic anti-inflammatory phase and establishment of the primary pneumonia [22]. In spite of the absence of hemoptysis and coagulopathy, the AGM model is suitable to test the efficacy of treatment for a bioterrorism-associated disease. In this study untreated AGMs exhibited multifocal pneumonia, high-grade bacteremia, dissemination to liver and spleen, and 100% lethality from 40 to145 aerosol-LD_50_ doses, confirming previous results [22]. The course of disease was rapid, with telemetry-documented fever onset occurring within 53–93 h of exposure in most animals. Three AGMs dosed below 40 LD_50_ doses had later onsets of fever of 124, 125, and 165 h post exposure, but in the study population as a whole time to onset of fever was not significantly related to inhaled dose of pathogen. Five of the 16 survivors randomized to treatment received an aerosol challenge less than 40 LD_50_ doses, but even if these 5 were removed from analysis, protection was still significant. Cynomolgus macaques are also highly susceptible to inhaled *Y. pestis* [35], and exhibit the two-phase disease course [33]. The macaques, however, may have the drawbacks of modest febrile response in some animals [33], and variable levels of innate resistance and unexpected survival following lethal doses [36] (K E Van Zandt et al, 2010, Efficacy of cethromycin against lethal *Yersinia pestis* inhalation challenge in cynomolgus macaques, Abstract B-057, 4th Biodefense Research, Am Soc Microbiol, Baltimore, MD).

### Initiation of treatment

Treatment infusions were initiated by the appearance of fever, and thus similar to the ‘late treatment’ studied in the mouse model [17]. The increase in body temperature detected by continuously monitored telemetry and defined as >39°C in all animals was in retrospect >2°C above the diurnal background (**Figure 3**). Bacteremia was found in most animals at the time of onset of fever. Chest radiographs taken 1–2 days after the onset of fever documented the presence of detectable pneumonia in all animals tested (Cohorts 1 and 2), consistent with the treatment of established pneumonia. Interestingly, one animal was removed from the study post-challenge due to premature treatment approximately 8 h *prior to* becoming febrile. Nonetheless, a single 8-mg/kg dose of levofloxacin which did not prevent fever resulted in survival for the 28-day observation period. Nonetheless, the important question remains unanswered how late in the progression of disease will levofloxacin remain efficacious in reversing the rapid progression of pneumonic plague.

### Antibiotic selection

Few antibiotics, including streptomycin and tetracyclines, are approved for the treatment of plague, but these antibiotics are toxic and have limited availability. Several antibiotics for plague, including doxycycline and gentamicin, have been supported by clinical experience [37], [38]. In the AGM model of pneumonic plague, however, oral doxycycline initiated within 6 h of the onset of fever resulted in only 40% survival (H Lockman et al. Efficacy of oral doxycycline against pneumonic plague in African Green monkeys. Abstract G-098, 4^th^ Biodefense Research Meeting, Am Soc Microbiol, Feb 22, 2010, Baltimore MD). An oral ketolide cethromycin has in vitro antimicrobial activity similar to gentamicin but in a cynomolgus macaque model of pneumonic plague the highest dose resulted in 90% survival when given only 24 h after inhalation challenge (K E Van Zandt et al, 2010, Efficacy of cethromycin against lethal *Yersinia pestis* inhalation challenge in cynomolgus macaques, Abstract B-057, 4th Biodefense Research, Am Soc Microbiol, Baltimore, MD). Many beta-lactam antibiotics and fluoroquinolones have significant in vitro activity against *Y. pestis* [39], but beta-lactam antibiotics did not have significant *in vivo* treatment efficacy whith late treatment in the mouse model [17]. Ciprofloxacin has been proposed as the primary treatment for mass casualties in a bioterrorism event involving *Y. pestis* [3]. In a post-exposure prophylaxis study following lethal aerosol challenge in mice, ciprofloxacin given 44h post-exposure was 90% effective in preventing death [40]. In the AGM ciprofloxacin showed efficacy for treatment of inhalational plague [Pitt MLM, DN Dyer, EK Leffel, et al. Ciprofloxacin treatment for established pneumonic plague in the African Green Monkey. Abstract B-576, 46^th^ICAAC meeting, San Francisco, CA, September 28, 2006]. In a post-exposure prophylaxis study for anthrax in rhesus macaques, however, ciprofloxacin did not prevent mortality in 56% of aerosol-challenged monkeys [41], leaving open the question of multi-biothreat agent efficacy of ciprofloxacin.

This study evaluated the efficacy of levofloxacin, a fluoroquinolone antibiotic with FDA approval for a wide range of Gram positive and Gram negative infections, including severe Gram negative pneumonias. This antibiotic has broad efficacy against many select agents including *Y. pestis* [42] and is used in most inpatient health care facilities in the United States, making it an appropriate candidate for rapid availability in the event of a bioterrorism event. Levofloxacin should continue as a candidate for such an event even in light of recently demonstrated toxicities [43], [44], which were not evaluated in this NHP model, due to the high and rapid morbidity of primary pneumonic plague. An evaluation has occured in an in vitro pharmacodynamic infection model levofloxacin sterilized the culture without resistance selection [45]. In a mouse study of pneumonic plague, levofloxacin treatment conferred 100% survival when treatment began 24 h after aerosol exposure to 20 LD_50_ doses [34]. Levofloxacin achieves high concentrations in human lung tissue and alveolar macrophages with levels 2–4 times that in plasma [46], [47].

### Antibiotic dosing

The human dose for Gram negative pneumonia is 500 mg intravenously every 24 h. The peak level in plasma following this dose is 6.2±1.0 µg/mL and an area under the curve of 48.3±5.4 µg·h/mL (Levaquin package insert), so these were our targeted levels. A previous study in rhesus macaques developed a “humanized” dosing regimen for levofloxacin, as the elimination of levofloxacin is 3-fold more rapid in nonhuman primates than humans [27]. Using data from an earlier study of levofloxacin pharmacokinetics in AGMs, the daily dose schedule used in this study was calculated to be 8 mg/kg followed by 2 mg/kg 12 h later (Blaire Osborn, unpublished data). Levofloxacin levels (**Table 1**) demonstrated that achieved levels were only half of the targeted C_max_, yet the dose administered was successful in curing established plague pneumonia. Nonetheless the interpretation of efficacy in this study is dependent on dosing sufficient to achieve a peak plasma dose of 2.4 µg/mL or greater, and continuous plasma levels above the minimum inhibitory concentration of the organism.

The standard of care for treatment of suspected bubonic plague instructs the inclusion of gentamicin among other broad-spectrum antibiotics [3], [37], [38]. Our results in the AGM model of pneumonic plague, the most severe of plague syndromes, suggests that levofloxacin would likely be efficacious in bubonic plague. There is no established model for bubonic plague in nonhuman primates but a clinical trial of levofloxacin could be safely undertaken for the treatment of bubonic plague.
